# Best clinical practice guidance for conscious sedation of children undergoing dental treatment: an EAPD policy document

**DOI:** 10.1007/s40368-021-00660-z

**Published:** 2021-08-28

**Authors:** P. Ashley, P. Anand, K. Andersson

**Affiliations:** 1 Paediatric Dentistry, UCL Eastman Dental Institute, Rockefeller Building, University St, London, WC1E 6DE UK; 2 Royal National ENT and Eastman Dental Hospitals, UCLH NHS Trust, 47-49 Huntley Street, London, WC1E 6DG UK; 3 Department of Dental Medicine, Division of Orthodontics and Pediatric Dentistry, Karolinska Institutet, POB 4064, SE-141 04 Huddinge, Sweden

**Keywords:** Sedation, Dental, Paediatric, Midazolam, Nitrous oxide

## Abstract

**Background:**

Due to fear and/or behaviour management problems, some children are unable to cooperate for dental treatment using local anaesthesia and psychological support alone. Sedation is required for these patients in order for dentists to be able to deliver high quality, pain-free dental care.

The aim of this guideline is to evaluate the efficacy and relative efficacy of conscious sedation agents and dosages for behaviour management in paediatric dentistry and to provide guidance as to which sedative agents should be used.

**Methods:**

These guidelines were developed using a multi-step approach adapted from that outlined by the National Institute for Clinical Excellence (NICE (2020) Developing NICE Guidelines: the manual. https://www.nice.org.uk/process/pmg20/chapter/introduction#main-stages-of-guideline-development. Accessed 7 Oct 2020). Evidence for this guideline was provided from a pre-existing Cochrane review (Ashley et al. Cochrane Database Syst Rev 12:CD003877, 2018) supplemented by an updated search and data extraction up to May 2020.

**Results:**

Studies were from 18 different countries and had recruited 4131 participants overall with an average of 70 participants per study. Ages ranged from 0 to 16 years with an average age of 5.6 years across all included studies. A wide variety of drugs or combinations of drugs (*n* = 38) were used and delivered orally, intranasally, intravenously, rectally, intramuscularly, submucosally, transmucosally or by inhalation sedation. Twenty-four different outcome measures for behaviour were used. The wide range of drug combinations and outcome measures used greatly complicated description and analysis of the data.

**Conclusion:**

Oral midazolam is recommended for conscious dental sedation. Midazolam delivered via other methods or nitrous oxide/oxygen sedation could be considered, but the evidence for both was very low.

**Supplementary Information:**

The online version contains supplementary material available at 10.1007/s40368-021-00660-z.

## Aim

The European Association of Paediatric Dentistry (EAPD) proposes this clinical guideline for practitioners wanting to use conscious sedation to support delivery of dental care in children and adolescents. The aim of this guideline is to evaluate the efficacy and relative efficacy of conscious sedation agents and dosages for behaviour management in paediatric dentistry and to provide guidance as to which sedative agents should be used. In addition, this guideline will provide a clinical protocol to guide dentists on the use of recommended dental sedative agents. This document replaces the former EAPD statement developed by Hallonsten et al. (2005) and incorporates the Cochrane review on sedation of children undergoing dental treatment (Ashley et al. 2018).

## Selection of the guidance topic

There are two main dimensions to paediatric oral care: (1) to keep the oral environment healthy, and (2) to keep the patient capable of, and willing to utilize the dental service. Maintenance of good oral health will often require operative intervention. Due to dental fear and/or dental behaviour management problems, some children may not be able to cooperate for treatment using local anaesthesia and psychological support alone. Treatment can be performed with delivery of general anaesthesia, however this should be avoided due to the associated need of specialist resources and potential risk of death (Ashley et al. 2018).

Sedation is an alternative for these child patients in order for dentists to be able to deliver high quality, pain-free dental care in a safe way without the need for general anaesthesia. It also has the potential to help the patient cope with continued use of paediatric dental services.

## Objectives for sedation in paediatric dentistry

Objectives for sedation in paediatric dental care consider both the needs of the child and the dentist:

### The child

- Reduce fear and perception of pain during the treatment
- Facilitate coping with the treatment
- Prevent development of dental fear and anxiety

### The dentist

- Facilitate accomplishment of dental procedures
- Reduce stress and unpleasant emotions

In recognition of the expanding need for both the elective and emergency use of sedative agents and the importance of delivering painless treatment to children, guidelines for the use of sedative agents among children are important.

Paediatric dentists should be aware that sedation represents a continuum. Thus, a patient may move easily from a light level of sedation to a deeper level, which may result in the loss of the patient’s protective reflexes. The distinction between conscious sedation and deep sedation is made for the purpose of describing the level of monitoring needed, as well as the responsibility of the dentist. Techniques that cannot be safely delivered by an operator/sedationist are unlikely to be ‘conscious’.

Conscious sedation can be defined as

- Minimally depressed consciousness
- Ability to maintain open airway
- Protective reflexes maintained
- Response to verbal and physical stimulation

This updated guideline will continue to only consider drugs and techniques that produce conscious sedation.

## Legislation, training and governance

The rules and regulations governing dental practice differ widely between European countries. Important differences as to the rights of the dentist to utilize various methods of sedation also exist. This guideline will present evidence-based recommendations on the efficacy of sedative agents and expert-based recommendations on appropriate training and governance for dentists practising sedation. These will need to be interpreted and used within the legislative framework of individual nation states.

## Education and training

Training of paediatric dentists in sedation should be theoretical and practical. EAPD Guidelines for postgraduate training in paediatric dentistry should be followed in developing appropriate training programmes in sedation (EAPD 1997).

Theoretical training should cover all the subjects referred to in the present document. Practical training should include knowledge of the drugs and equipment used for conscious sedation and must be completed before the clinical training. Knowledge of management of complications due to conscious sedation is essential. Training and experience should be regularly updated and maintained.

Documented, contemporaneous supervised hands-on experience must be acquired for each conscious sedation technique used. The minimum number of documented supervised cases completed should be no less than those specified by appropriate authorities.

Dental auxiliary personnel assisting during conscious sedation sessions shall also have appropriate training.

All clinical staff require theory and practical training in basic life support. Basic life support must conform to contemporary guidelines issued by national authorities and dental associations. Training can be through informal courses where clinical training is included or in theoretical courses with clinical demonstrations in combinations with clinics where conscious sedation is regularly performed for hands-on supervision.

Those arranging such training have a duty to ensure that the quality of training and trainers is appropriate, and that all theoretical and practical training is documented.

## Conscious sedation agents and dosages for behaviour management in paediatric dentistry in children up to the age of 16 years

### Methodology

These guidelines were developed using a multi-step approach adapted from that outlined by the National Institute for Clinical Excellence (NICE 2020). Input from children and young people was not taken into account in this review. Resource implications were also not considered.

A draft revision of the guideline was written by the authors. We started the process from the evidence review stage. Moving from evidence to draft recommendations was undertaken following the GRADE methodology (Guyatt et al. 2008). This was then submitted to the Clinical Advisory Group of the European Association of Paediatric Dentistry before presentation to the membership at the EAPD Interim meeting in 2021.

### Evidence review

Evidence for this guideline was provided from a pre-existing Cochrane review (Ashley et al. 2018) supplemented by an updated search and data extraction (up to May 2020). Details of the methodology can be found in the pre-existing Cochrane review. A summary of the evidence review methodology follows.

### Selection criteria and types of studies

Studies were selected if they met the following criteria: randomised controlled trials of conscious sedation comparing two or more drugs/techniques/placebo undertaken by the dentist or one of the dental team in children up to 16 years of age. Quasi-randomised trials were excluded. We also excluded cross-over trials from this review, as they are not an appropriate study design when the intervention can have a long-lasting effect (Higgins 2011) or for studies investigating the efficacy of sedative agents (Gomes et al. 2019).

### Search strategy

Searches were carried out by the Cochrane Oral Health Information Specialist in the following databases (up to 22 Feb 2018). There were no language or publication status restrictions. Details of the search strategy are reported in the Cochrane review.

- Cochrane Oral Health Trials Register
- Cochrane Central Register of Controlled Trials
- Medline OVID
- Embase OVID

The search was updated for this review by the information specialist at the Karolinska Institutet using the same search strategy. The following databases were searched up to 20 May 2020.

- Medline OVID
- Embase OVID

Risk of Bias was assessed using Cochrane’s risk of bias tool (Higgins 2011).

### Data synthesis

Outcomes considered to be most important to the aim of this review were

- Completion of treatment (yes/no)
- Difference in behaviour between test and control groups
- Difference in post-operative anxiety between test and control groups
- Adverse events

Trials included in the review presented with complex data, very different interventions and a wide range of outcome measures. Therefore to aid description and analysis we separated studies into three groups:

- Active treatment vs placebo
- Different doses of the same agent
- Different agents vs each other

Data were predominantly presented in a narrative format as there were few options to combine data into a meta-analysis.

The certainty of the evidence was assessed using GRADE methodology. We produced 'Summary of findings' tables for the main comparisons of the review and the following outcomes: mean Houpt/other behavioral score and good or better behaviour, and adverse events. We used GRADE methods and the GRADEpro online tool for developing the 'Summary of findings' tables (www.guidelinedevelopment.org). We assessed the certainty of the body of evidence for each comparison and outcome by considering the overall risk of bias of the included studies, the directness of the evidence, the inconsistency of the results, the precision of the estimates, and the risk of publication bias. We categorised the certainty of each body of evidence as high, moderate, low, or very low. Economic factors were not considered.

### Clinical evidence

Results of the search are summarised below, data from the original Cochrane review and the updated search have been combined. More detailed description of the original data can be found in the Cochrane review (Ashley et al. 2018), detailed results from the updated search can be found in the supplements to this guideline as indicated below.

### Results of the search

Six additional studies were included in the review and added to those studies already identified in the Cochrane review bringing the total number to 56 (Prisma flowchart of the search results and excluded studies is in Supplement 1). Studies were from 18 different countries with India being the most common (*n* = 16, 18%). Studies had recruited 4131 participants overall all with an average of 70 participants per study. Ages ranged from 0 to 16 years with an average age of 5.6 years across all included studies. Characteristics of the included studies from the updated search are in Supplement 2.

A wide variety of drugs or combinations of drugs (*n* = 38) were used and delivered orally, intranasally, intravenously, rectally, intramuscularly, submucosally, transmucosally or by inhalation sedation. Supplemental nitrous oxide/oxygen in combination with a papoose board was used in 23% of the studies. Dental treatment was poorly described on the whole. Drugs recorded from the updated search are in Supplement 3.

Most of the included papers did not state explicitly whether they were practicing conscious or deep sedation, sleeping was also poorly reported. We believe that in some of these papers deep sedation was undertaken, as participants were reported as falling asleep and mouth props were used.

Sequence generation and allocation concealment were generally poorly reported and often scored as unclear. Nine of the studies (16%) had no blinding at all, in three (5%) it was unclear and in seven (13%) only the outcome assessor was blinded. Risk of bias assessments of the studies included from the updated search are in Supplement 4.

A range of outcome measures were originally proposed for this review however meaningful data could only be collected for behaviour. Completion of treatment was not used as in most studies both arms successfully completed treatment. Twenty-four different outcome measures for behaviour were used. The efficacy of a particular agent will be influenced by the baseline anxiety of the child involved. Ideally this should always be recorded and then compared to levels of anxiety after sedation. Baseline values of anxiety were not uniformly reported and very few studies recorded anxiety at the end. Outcome measures recorded from the updated search are in Supplement 5.

The wide range of drug combinations and outcome measures used greatly complicated description and analysis of the data. Therefore, studies were separated into three categories:

- Studies where test drug(s) were compared to a placebo.
- Studies where differing dosages of the same drug(s) were compared.
- Studies comparing different drugs or combinations of drugs.

### Placebo studies

There were 12 placebo studies which looked at oral chloral hydrate, intranasal dexmedetomidine, oral diazepam, melatonin, intramuscular meperidine, oral midazolam, intravenous midazolam, midazolam/ketamine and nitrous oxide (Table 1). No additional studies were found in the updated search.

**Table 1:** Summary of Findings; sedative compared to placebo for children needing dental care

Patient or population: children needing dental care Setting: hospital Intervention: sedative Comparison: placebo						
Outcomes	Anticipated absolute effects (95% CI)		Relative effect (95% CI)	Number of participants (studies)	Certainty of the evidence (GRADE)	Comments
	Risk with Placebo	Risk with Sedative				
Houpt/other behavioural score—Midazolam (oral) SD units: investigators measure behaviour using different scales—Higher values mean better behaviour	The Houpt/other behavioural score in the midazolam (oral) group was on average 1.96 SDs higher (1.59 higher to 2.33 higher) than the placebo group			202 (6 RCTs)	⊕ ⊕ ⊕ ⊝ MODERATE^1^	As a rule of thumb 0.2 SD represents a small difference, 0.5 a moderate difference, and 0.8 a large difference Adverse events: vomiting/hiccupping reported in one study. Amnesia reported in one study Oral midazolam probably improves behaviour
Houpt/other behavioural score—Midazolam (intravenous) SD units: investigators measure behaviour using different scales—Higher values mean better behaviour	The Houpt/other behavioural score in the midazolam (intravenous) group was on average 1.21 SDs higher (0.24 higher to 2.18 higher) than the placebo group			20 (1 RCT)	⊕ ⊝ ⊝ ⊝ VERY LOW^1, 2^	As a rule of thumb 0.2 SD represents a small difference, 0.5 a moderate difference, and 0.8 a large difference No adverse events reported Uncertain whether intravenous midazolam improves behaviour
Houpt/other behavioural score—Nitrous oxide SD units: investigators measure behaviour using different scales—Higher values mean better behaviour	The Houpt/other behavioural score in the nitrous oxide group was on average 0.69 SDs higher (0.13 higher to 1.26 higher) than the placebo group			52 (1 RCT)	⊕ ⊝ ⊝ ⊝ VERY LOW^1, 3^	As a rule of thumb 0.2 SD represents a small difference, 0.5 a moderate difference, and 0.8 a large difference No adverse events reported Uncertain whether nitrous oxide improves behaviour
Houpt/other behavioural score—Diazepam (oral) SD units: investigators measure behaviour using different scales—Higher values mean better behaviour	The Houpt/other behavioural score in the diazepam (oral) group was on average 0.62 SDs higher (0.28 lower to 1.53 higher) than the placebo group			20 (1 RCT)	⊕ ⊝ ⊝ ⊝ VERY LOW^1, 2^	As a rule of thumb 0.2 SD represents a small difference, 0.5 a moderate difference, and 0.8 a large difference No adverse events reported Uncertain whether oral diazepam improves behaviour
Good or better behaviour—Chloral hydrate	Study population		RR 1.33 (0.80–2.22)	60 (1 RCT)	⊕ ⊝ ⊝ ⊝ VERY LOW^3, 4^	Adverse events: associated with airway problems Uncertain whether chloral hydrate improves behaviour
	533 per 1000	709 per 1000 (427–1000)				
Good or better behaviour—Meperidine	Study population		RR 5.33 (1.45–19.64)	60 (1 RCT)	⊕ ⊕ ⊝ ⊝ LOW^5^	Adverse events: nausea, vomiting and unmanageable behaviour were associated with meperidine use Meperidine may improve behaviour
	133 per 1000	711 per 1000 (193–1000)				

GRADE Working Group grades of evidence

High certainty: we are very confident that the true effect lies close to that of the estimate of the effect

Moderate certainty: we are moderately confident in the effect estimate: the true effect is likely to be close to the estimate of the effect, but there is a possibility that it is substantially different

Low certainty: our confidence in the effect estimate is limited: the true effect may be substantially different from the estimate of the effect

Very low certainty: we have very little confidence in the effect estimate: the true effect is likely to be substantially different from the estimate of effect

*CI* confidence interval; *RCT* randomised controlled trial; *RR* risk ratio; *SD* standard deviation; *SMD* standardized mean difference

*The risk in the intervention group (and its 95% CI) is based on the assumed risk in the comparison group and the relative effect of the intervention (and its 95% CI)

^1^Downgraded for risk of bias (lack of blinding and randomisation processes unclear)

^2^Downgraded for imprecision (large confidence interval and small numbers)

^3^Downgraded for imprecision (large confidence interval)

^4^Downgraded for risk of bias (randomisation unclear and incomplete outcome assessment)

^5^Downgraded for risk of bias (randomisation unclear) and imprecision

### Dosage comparison studies

There were 11 studies which compared different dosages or routes of admission of sedative agents: one used hydroxyzine, one looked at different dosages and methods of delivering dexmedetomidine (Patel et al. 2018, added from updated search), the remaining nine varied dosage or method of midazolam with six primarily using intranasal midazolam and three oral midazolam (Table 2). Data from studies from the updated search are in Supplement 6.

**Table 2:** Summary of findings: Sedative compared with different dosage (or method application) of the same sedative for children needing dental care

Patient or population: children needing dental care Setting: hospital Intervention: sedative Comparison: placebo			
Outcomes	Number of participants (studies)	Certainty of the evidence (GRADE)	Comments
Any behavioural score Midazolam (any mode of delivery)	394 (10)	⊕ ⊝ ⊝ ⊝ VERY LOW^1^	There is insufficient evidence to determine whether any specific dose of intranasal midazolam is effective There is weak evidence from two trials that oral midazolam at a dose of 0.5–0.75 mg/kg is an effective sedative for children. However, one trial administered both nitrous oxide and midazolam so it is difficult to attribute benefit to midazolam alone
Any behavioural score Hydroxyzine	30 (1)	⊕ ⊝ ⊝ ⊝ VERY LOW^1^	There is insufficient evidence to determine whether any specific dose of hydroxyzine is effective
Any behavioural score Dexmedetomidine	44 (1)	⊕ ⊝ ⊝ ⊝ VERY LOW^1^	There is insufficient evidence to determine whether any specific dose of dexmedetomidine is effective or whether intranasal administration is more or less effective than oral administration

GRADE Working Group grades of evidence

High certainty: we are very confident that the true effect lies close to that of the estimate of the effect

Moderate certainty: we are moderately confident in the effect estimate: the true effect is likely to be close to the estimate of the effect, but there is a possibility that it is substantially different

Low certainty: our confidence in the effect estimate is limited: the true effect may be substantially different from the estimate of the effect

Very low certainty: we have very little confidence in the effect estimate: the true effect is likely to be substantially different from the estimate of effect

^1^Downgraded for risk of bias, inconsistency and/or imprecision

### Drug comparison studies

There were 35 studies that compared different drugs or combinations of drugs which are summarised in Table 3. Five of these were added following the updated search with one comparing IV dexmedetomidine with ketamine/atropine, one comparing IV ketamine with IV propofol and IV ketamine and propofol, three looking at oral midazolam/ketamine compared to either nitrous oxide/oxygen, dexmedetomidine/fentanyl, dexmedetomidine/ketamine, midazolam (oral) or intranasal midazolam ketamine).

**Table 3:** Summary of findings: Sedative compared with a different sedative for children needing dental care

Patient or population: children needing dental care Setting: hospital Intervention: sedative Comparison: placebo			
Outcomes	Number of participants (studies)	Certainty of the evidence (GRADE)	Comments
Any behavioural score Chloral hydrate/hydroxyzine versus	235 (6)	⊕ ⊝ ⊝ ⊝ VERY LOW^1^	Very few studies evaluated the same intervention and comparisons. No studies that did evaluate similar interventions and comparisons found the same effect. There is insufficient evidence to draw any conclusions
Any behavioural score Chloral hydrate/promethazine versus	24 (1)	⊕ ⊝ ⊝ ⊝ VERY LOW^1^	
Any behavioural score Dexmedetomidine versus	160 (3)	⊕ ⊝ ⊝ ⊝ VERY LOW^1^	
Any behavioural score Ketamine vs	569 (9)	⊕ ⊝ ⊝ ⊝ VERY LOW^1^	
Any behavioural score Ketamine/midazolam vs	175 (4)	⊕ ⊝ ⊝ ⊝ VERY LOW^1^	
Any behavioural score Midazolam (oral) vs	654 (7)	⊕ ⊝ ⊝ ⊝ VERY LOW^1^	
Any behavioural score Midazolam (intravenous) vs	70 (2)	⊕ ⊝ ⊝ ⊝ VERY LOW^1^	
Any behavioural score Midazolam (rectal) vs	90 (1)	⊕ ⊝ ⊝ ⊝ VERY LOW^1^	
Any behavioural score Sevoflurane vs	1140 (3)	⊕ ⊝ ⊝ ⊝ VERY LOW^1^	

GRADE Working Group grades of evidence

High certainty: we are very confident that the true effect lies close to that of the estimate of the effect

Moderate certainty: we are moderately confident in the effect estimate: the true effect is likely to be close to the estimate of the effect, but there is a possibility that it is substantially different

Low certainty: our confidence in the effect estimate is limited: the true effect may be substantially different from the estimate of the effect

Very low certainty: we have very little confidence in the effect estimate: the true effect is likely to be substantially different from the estimate of effect

^1^Downgraded for risk of bias, inconsistency and/or imprecision

### Adverse effects

There is insufficient evidence from trials in this review to support the effectiveness of either chloral hydrate or ketamine. However, it should be noted that chloral hydrate was associated with significant adverse effects, specifically airway issues especially when high doses (> 50 mg/kg) were combined with the use of inhalational nitrous oxide. Ketamine was also associated with significant adverse effects.

### Draft clinical recommendations

Due to the poor quality of data from both the drug comparison groups and dosage groups, we decided not to use these to develop recommendations.

### Strong

We recommend oral midazolam for sedation of children needing dental treatment.

#### *Remarks*

Oral midazolam for dental sedation in children is supported by moderate quality evidence and at appropriate dosages is safe to use and acceptable to children. This review did not consider whether or not placing an IV cannula for delivery of flumazenil was required when giving oral midazolam.

### Weak

We suggest nitrous oxide/oxygen is used for sedation of children needing dental treatment.

#### *Remarks*

Nitrous/oxide oxygen only has very low quality evidence supporting its use. We have recommended it as a conscious sedation agent due to its well-known anxiolytic and sedative effects combined with rapid onset and recovery and its overall safety. It is already in widespread use in dentistry and medicine as a sedative agent for children and adults.

We suggest midazolam delivered by IV or any transmucosal route is used for sedation of children needing dental treatment.

#### *Remarks*

Midazolam delivered by IV or any transmucosal route only has very low-quality evidence supporting its use. We have recommended it as a conscious sedation agent due to its well-known anxiolytic and sedative effects combined with rapid onset and recovery and its overall safety. It is already in widespread use in dentistry and medicine as a sedative agent for children and adults.

Chloral hydrate and meperidine were not recommended as agents for the dental sedation of children because of the risk of adverse events. Diazepam was not recommended as an agent for the dental sedation of children as compared to midazolam it has high tissue solubility, prolonged elimination time (24–48 h) and active metabolites. It may be better for preoperative anxiolysis the day before surgery rather than on the day of the operative procedure.

## Research recommendations

### Develop a core outcome set to measure dental sedation effectiveness in children

#### Rationale

Currently a large number of different outcomes are used in studies investigating dental sedation making comparison between studies difficult or impossible. They are predominantly clinician centred and focussed on the ease of treatment provision. They are often based on movement or obvious signs of distress, therefore may have little value in studies where children are restrained or heavily sedated. Patient satisfaction, reduction in anxiety or other patient centred measures are rarely used. Demographic variables are often incompletely reported. Depth of sedation is unclear and definitions of conscious or deep sedation are inconsistently applied (if used at all). Use of restraint or adjunctive nitrous oxide/oxygen is unclear in some studies.

### Investigate the effectiveness of new dental sedation agents by comparing to a placebo or widely used reference technique

#### Rationale

Drugs were commonly compared to other combinations of drugs that themselves had no significant evidence base. New drugs or drug combinations for conscious dental sedation should be tested against standard and commonly used techniques.

### Investigate the effectiveness of dental sedation in reducing dental anxiety

#### Rationale

Dental sedation could be used to facilitate the introduction of treatment to anxious children with a view to reducing or removing sedation in subsequent visits (an approach taken by Veerkamp 1993).

### Investigate the effectiveness of dental sedation in different age groups

#### Rationale

The majority of studies involved sedation in children less than 6 years of age, probably because this age range belongs to a 'pre-co-operative' group. Treatment needs and management of children will vary as they grow and develop. Techniques that are appropriate in a 3-year-old may not be appropriate in a 12-year-old and vice versa.

## Using midazolam and nitrous oxide—clinical protocol

### Patient selection and assessment

Patient assessment must include a full medical, dental and social history. Each patient should be classified according to the ASA Physical Status Classification System (ASA 1963). Patients who are ASA Class I or Class II may be considered candidates for conscious sedation as outpatients. Patients in ASA Class III and Class IV represent special problems requiring individual consideration and are best treated in a hospital environment. Medical colleagues should be consulted where appropriate.

### Indications and contraindications

A combined consideration of the following two groups of factors may be appropriate for identification of children in need of conscious sedation.

1. Children unable to cope, e.g., dental anxiety, special needs.
2. Treatment required, e.g., emergency or large volume.

Sedation of children below the age of 1 year or < 10 kg is hardly ever relevant in the dental setting and should not be performed without consulting with an anaesthesiologist (Kapur and Kapur 2018). Conscious sedation during pregnancy requires careful assessment of risks versus the benefits and represents a relative contraindication to extensive elective dental care, particularly during the first trimester.

### Patient information

#### Written and oral information and consent

The child and the parent or guardian should have oral and written pre- and postoperative instructions in advance of the procedure. Informed consent should follow the legislation of the country. The child should always be escorted to and from treatment by a responsible adult who is well known to the child. Provided the parents have consented, schoolchildren can get treatment with nitrous oxide/oxygen without the presence of an adult escort in the context of school dental clinics.

### Patient monitoring

#### Continuous clinical observation

Paediatric dental patients under conscious sedation must be monitored continuously clinically, as this is the most important element in patient monitoring. Clinical monitoring can include:

- Level of consciousness/depth of sedation
- Airway patency
- Observe breathing including movements of the thorax
- Respiraton-rate and depth
- Observing skin colour
- Pulse rate, rhythm and volume
- Adequate pain control including adequate local anaesthesia

The clinical team must be able to recognise a deteriorating patient and manage accordingly. It is vital that staff are adequately trained in the use of clinical monitoring and electronic monitoring. Any electronic monitoring used must be age appropriate.

#### Pulsoximetry and blood pressure monitoring

In the case of conscious sedation, oxygen desaturation below 95% in children is rare. Nevertheless the use of pulse oximetry has been widely discussed. Pulsoximetry and blood pressure monitoring is not usually deemed necessary for conscious sedation with nitrous oxide/oxygen but is normally expected for benzodiazepine sedation. When pulse oximetry is used, the alarms may show false positive due to movement artefacts, sensor displacement or other reasons. Young children especially may react with increased anxiety to the placement of the pulsoximeter.

### Fasting

Fasting prior to sedation continues to be a controversial topic and each country’s legislation for this must be followed according to the local guidance. There is only low evidence available for this (NICE 2010; IACSD 2015; SDCEP 2017). For conscious sedation, an individual assessment needs to be made on the basis of the dental procedure, patient’s medical assessment and the sedation technique being used. Depending on the circumstances, it may or may not be appropriate for the patient to modify food and drink intake before sedation.

After carefully considering all factors for a patient:

- If the decision is to not fast, a patient should be advised that although they can eat and drink on the day, they need to avoid alcoholic drinks and large meals.
- If there is a significant risk of aspiration, or another indication, consider fasting prior to sedation. The 2–4–6 fasting rule is recommended in this situation.

It is advisable to confirm and record food and fluid intake on the day of sedation.

### Discharge

The recovery of a child must be assessed before discharge. At time of discharge, the patient should be alert and oriented (or have returned to an age-appropriate base line). A responsible adult must be present to observe the child for complications after discharge. In case of midazolam sedation, this adult should ensure that the child is in a position to facilitate breathing. If the responsible adult is driving, another adult must be present if the child is young.

The adult must be given written and oral instructions not only related to the sedation technique, but also the dental procedure conducted including appropriate diet, medications, level of activity and management of possible postoperative bleeding.

### Documentation and records

It is recommended that the documentation include

- Medical history including prescribed medication
- Previous dental history
- History of previous conscious sedations and general anaesthesia
- Indication for the use of conscious sedation
- Pre-sedation assessment
- Written instructions provided pre- and post-operatively
- Presence of an accompanying responsible adult
- Arrangements for suitable post-operative transportation and supervision
- Compliance with pre-treatment instructions
- The course of the treatment
- Monitoring
  - Dose, and route of administration of sedative drugs of Dental treatment performed
  - Sedation evaluation (sedation scale)
  - Acceptance of sedation and treatment (behavioural scale)
  - Complications
- Post-sedation assessment and time of discharge home

Some examples of possible scales that could be used to monitor the effect of the sedation are in Table 4.

**Table 4:** Recommended Sedation records during and after sedation according to Wilson et al. (1990) and DSTG (2020)

Sedation techniques	
IV	Intravenous sedation
RA	Inhalation sedation
O	Oral sedation
TM	Transmucosal

Sedation scoring	
1	Fully awake and orientated
2	Drowsy
3	Eyes closed, responds promptly on verbal command
4	Eyes closed, rousable on mild physical stimulus
5	Eyes closed, unrousable on mild physical stimulus

Assessment of operating conditions		
1	Good	Patient fully cooperative with optimum degree of sedation
2	Fair	Minimal interference from patient due to over/under sedation
3	Poor	Operating difficult due to over/under sedation
4	Impossible	Action taken (e.g., GA)

Recovery	
Normal	Within the timescale expected
Rapid	Sooner than normal—action taken
Prolonged	Longer than normal—action taken

### Using Nitrous oxide

Nitrous oxide is a gas with anxiolytic and sedative effects combined with varying degree of analgesia and muscular relaxation. It has been suggested that both GABA a and NMDA- receptors are affected by nitrous oxide (Jevtovic-Todorovic et al. 1998; Fujinaga and Maze 2002; Sanders et al. 2008). To safeguard the patient’s oxygen supply during inhalation, nitrous oxide must be given in a mixture with oxygen (> 30%). Nitrous oxide is non-irritant to the respiratory tract, has a low tissue solubility, and a minimum alveolar concentration (MAC) value of more than one atmosphere. Therefore, nitrous oxide has an onset and recovery within minutes, and is a poor anaesthetic.

#### Indications

Nitrous oxide/oxygen sedation is useful in children who can cope with nasal breathing instructions, often 3 years and older.

Further to the general indications for conscious sedation mentioned previously, nitrous oxide/oxygen can be used in patients with a strong gag reflex, as well as in patients with muscular tone disorders such as cerebral palsy, to avoid unintentional movements.

Patients belonging to ASA Class III and Class IV can be treated with the help of nitrous oxide/oxygen sedation provided other indications are present, but treatment of these patients should be in conjunction with responsible medical colleagues and in a hospital setting.

#### Contraindications

Nitrous oxide/oxygen sedation should not be used in

- Pre-co-operative children
- Patients with upper airway problems as common cold, tonsillitis, sinusitis or nasal blockage
- Middle ear infection
- Patients with sinusitis or recent ENT operations (within 14 days)
- Patients in bleomycin chemotherapy
- Severe emotional or drug-related dependencies
- Chronic obstructive pulmonary disease
- Raised intraocular pressure, retinal surgery, intestinal obstructive surgery
- Untreated B12 deficiency (Stach 1995; Haas 1999; AAPD 2018)

Whenever possible and appropriate, medical specialists should be consulted before administering nitrous oxide to patients with significant underlying medical conditions.

#### Adverse effects

Observed side effects of nitrous oxide are over sedation, nausea, vomiting, sweating, dysphoria, restlessness, panics and headache (Jastak 1975; Hallonsten 1982; Veerkamp 1990).

#### Technique

Sedation is initiated by inhalation of pure oxygen for 2–5 min. Following that, the nitrous oxide concentration is gradually increased every second minute. The maximum recommended concentration of nitrous oxide is determined by national regulations, and varies between the Europe countries from 50 to 70%. The commonly effective dosage for most children tends to be 30–40%. At the end of the session the child is given pure oxygen for 5 min before discharge.

#### Potential interactions

Nitrous oxide may amplify the effects of other sedatives, e.g., opioids, benzodiazepines, leading to CNS depression. There are no known potential interactions with other drugs.

#### Safety for the staff

Chronic exposure to certain environmental concentrations of nitrous oxide has been reported to constitute an health risk for the dental staff (Rowland et al. 1992, 1995; Zaffina et al. 2019). Consequently, the dental staff must follow strict indications for the use of nitrous oxide, only use nitrous oxide delivery systems with an efficient scavenging system, have appropriate technique for disconnection of the delivery system, and have methods for testing the integrity of the breathing system.

### Using midazolam

Midazolam is a short-acting benzodiazepine with rapid onset of action. It has anxiolytic, sedative, hypnotic, anticonvulsant and muscle relaxant activity and frequently induces anterograde amnesia. Midazolam binds to the benzodiazepine receptor in the CNS and enhances the inhibitory action of the neurotransmitter GABA. The inhibitory effect of GABA is caused by increasing the flux of chloride ions through the ion channels of the nerve cell. The increase of chloride ions into the cell decreases its ability to initiate an action potential (Nordt and Clark 1997).

#### Indications

See general indications for sedation. Where moderate sedation as opposed to only mild sedation is required.

#### Contraindications

Midazolam must not be given to the following groups of children

- Children under the age of 1 year or body weight < 10 kg
- Children with any form of acute disease
- Children with respiratory or cardiac disease that affects daily life
- Children with neuromuscular diseases as myasthenia gravis
- Children with allergy to BZD
- Children with sleep apnoea
- Children with liver dysfunction (dose adjustment may be necessary)
- Children with hepatic dysfunction (dose adjustment may be necessary)
- Children with porhyria

Whenever appropriate, medical specialists should be consulted before administering midazolam to patients with significant underlying medical conditions (e.g., cardiac, pulmonary, kidney or liver dysfunction or current medication with centrally acting analgesics).

#### Adverse effects

The following side effects have been noted:

- Hiccups
- Nausea
- Respiratory depression
- Interactions with other medication
- Paradoxical reaction
- Over sedation
- Hallucinations

#### Clinical considerations

All drugs in use in the treatment area must be clearly labelled and each drug should be given according to accepted recommendations.

Flumazenil should be available in case needed to reverse the effects of midazolam in an emergency.

#### Routes

- Oral midazolam can be administered as a sweetened mixture for delivery either in a cup or drawn into a needleless syringe and deposited in the retromolar area. A preformulated flavoured syrup is also available for use.
- Intravenous midazolam can be administered via a cannula directly into circulation and titrated to affect.
- Transmucosal administration (rectal and intranasal) of midazolam has the advantage of depositing the drug directly into the systemic circulation.
- Rectal administration requires syringes and a rectal applicator. In some countries, rectal administration is uncommon due to cultural attitude.
- Intranasal sedation can be sprayed into one nostril.

#### Dosage

##### Oral

Children under 25 kg of weight shall have 0.3–0.5 mg midazolam per kilogram bodyweight. Maximum dose: 10–12 mg based on local legislation.

Children over 25 kg of weight shall have 10–12 mg midazolam based on local legislation.

Oral mixtures are given approximately 15–20 min before. The duration of effect is usually 30–50 min.

##### Rectal

Children under 25 kg of weight shall have 0.3–0.4 mg midazolam per kilogram bodyweight. Maximum dose 10 mg midazolam.

Children over 25 kg of weight shall have 10 mg midazolam.

Rectal solution is administered approximately 10 min before treatment starts.

##### Intranasal

Intranasal sedation can be sprayed into one nostril, using mucosal atomizer device (MAD)**.**

Formulation made up for this purpose needs to be quite concentrated to allow a small volume to be effective while being deposited in the nostril.

Dosage 0.2 mg/kg, maximum dose 10 mg.

The effect of the sedation takes place in approximately 10–15 min. The duration of effect is usually around 30 min.

##### Intravenous

1 mg initial loading dose over 60 s followed by 60 s increment of 1 mg until patient is ready for treatment. Dosage usually ranges from 2 to 7.5 mg. The effect of the sedation takes place in 1–2 min. The duration of effect is usually 30–50 min.

The effect of sedation may exhibit an interpersonal and intrapersonal variation.

##### Potential interactions

Contemporaneous intake of erythromycin, hypnotics, anxiolytics, antidepressants, some antifungals, some antivirals, antipsychotics, antiepileptics, antihistamines, opioids, grapefruit juice, clonidine and alcohol can enhance the effect. Drug interactions should be followed by the practitioner in their respective national databases.

### Flumazenil–midazolam antidote

Flumazenil is a selective GABA receptor antagonist. It acts as an antagonist and antidote to benzodiazepines through competitive inhibition. It is mostly administered intravenously for rapid onset and reversal of effects of benzodiazepines like midazolam.

The elimination half-time of Flumazenil is however shorter than that of midazolam. Hence patients must be carefully monitored to prevent recurrence of overdose symptoms. Repeat doses of flumazenil may be required for this reason too.

- Recommended dose of flumazenil (in child > 1 yr) given as intravenous administration: 10 µgm/kg, up to 200 µgm, over 15 s
- Repeat every 1 min × 4, Max 1 mg, i.e., two ampoules of 500 µgm or 50 µgm/kg, whichever is less
- 5 yr, 20 kg child; max 1000 µgm (2 amps)
- 12 yr, 40 kg child; max 1000 µgm (2 amps)

### Mandatory equipment for emergency situations during sedation with midazolam

Mandatory equipment as would be required by legislation of the country should include all equipment for a medical emergency, which would include age appropriate.

- Oxygen equipment
- Ventilation mask
- Pulsoximeter

## Supplementary Information

Below is the link to the electronic supplementary material.

Supplementary file1 (PDF 242 KB)

## Data Availability

Not applicable.
