# Supplementary material for: Best clinical practice guidance for conscious sedation of children undergoing dental treatment: an EAPD policy document
Source: Eur Arch Paediatr Dent. 2021 Aug 28;22(6):989–1002. doi: 10.1007/s40368-021-00660-z (PMC8629790; doi:10.1007/s40368-021-00660-z)
Supplement: Supplementary file 1 — Supplementary file1 (PDF 242 KB) [file 40368_2021_660_MOESM1_ESM.pdf]

Best clinical practice guidance for conscious sedation of children undergoing dental treatment: an EAPD policy document

European Archives Paediatric Dentistry

Paul Ashley 1
Prabhleen Anand 2
Kristofer Anderson 3

1 UCL Eastman Dental Institute, London
2 Royal National ENT and Eastman Dental Hospitals, London
3 Karolinska Institutet, Stockholm

Address for Correspondence
P Ashley
Paediatric Dentistry
UCL Eastman Dental Institute
Rockefeller Building
University St
London
WC1E 6DE

p.ashley@ucl.ac.uk

article title, journal name, author names; affiliation and e-mail address of the corresponding author.

Supplement 1 PRISMA 2009 Flow Diagram[1]

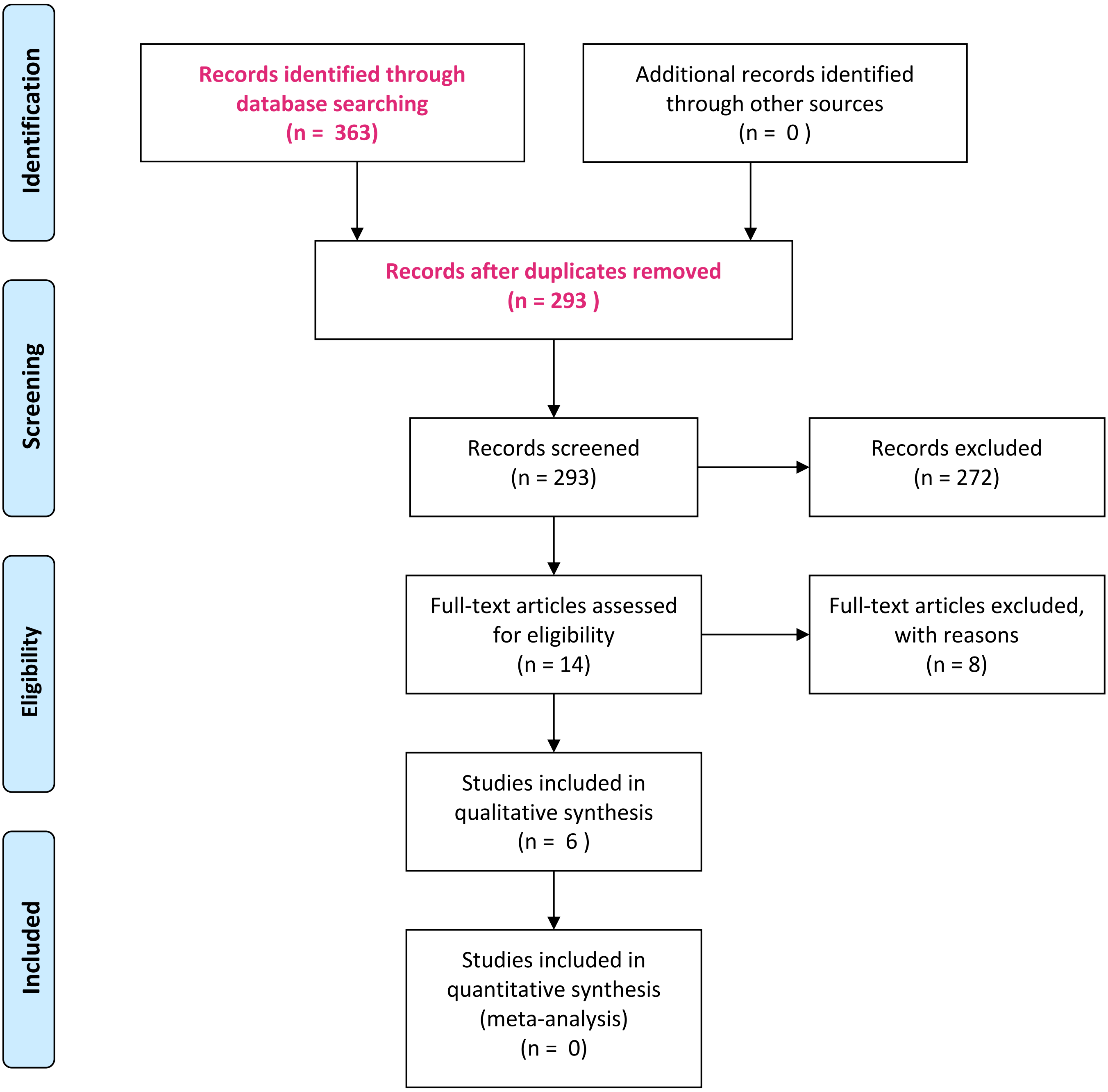

[1] From: Moher D, Liberati A, Tetzlaff J, Altman DG, The PRISMA Group (2009). Preferred Reporting Items for Systematic Reviews and Meta-Analyses: The PRISMA Statement. PLoS Med 6(6): e1000097. doi:10.1371/journal.pmed1000097. For more information, visit www.prisma-statement.org.

Excluded studies

Study	Reason for exclusion
Dixon 2020	Not an RCT
Hao 2017	Surgical removal of impacted supernumerary teeth only
Khalil 2020	Looking at effect of lignocaine on acceptance of midazolam. No data on sedation
Kharouba 2020	Not an RCT
Kip 2018	General anaesthesia, induced with sevoflurane
Lin 2019	Not an RCT
Mahdavi 2018	Crossover

Supplement 2 PRISMA 2009 Flow Diagram

Characteristics of included studies from updated search

Hammadyeh 2019

Methods

Parallel design
Funding: University
Location: Syria

Participants

Inclusion criteria: negative and definitely negative criteria on Frankl scale
N= 40
Age range 2-6
Mean age (SD) in years, gender, weight (SD) in kg
Group 1 9.1 (0.9), 10 males and 10 females, 14.9 (9.1)
Group 2 8.9 (0.7), 9 males and 11 females, 15.2 (8.2)

Interventions

Group 1 (n=20): dexmedetomidine loading dose of 1 μg/kg over 10 min, followed by 0.2 μg/kg as continuous infusion until desired level of sedation achieved
Group 2 (n=20): ketamine hydrochloride 2 mg/kg + atropine 0.01 mg/kg
All IV

Outcomes

Ohio State University Behavioral Rating Scale

Risk of Bias

Bias	Authors' judgement	Support for Judgement
Random sequence generation (selection bias)	Low risk	Computer generated
Allocation concealment (selection bias)	High risk	No concealment
Blinding - Participant	High risk	No blinding
Blinding - Operator/sedationist	High risk	No blinding
Blinding - Outcome assessor	Unclear risk	Not described
Incomplete outcome assessment	Low risk	All randomised participants included in outcome assessment
Free of selective reporting	Low risk	All planned outcomes reported
Free of other bias	Unclear	No apparent differences between groups at baseline however mean age is stated as 9 but the range is 2-6

Ilasrinivasan 2018

Parallel design
Funding: None
Location: India

Participants
Inclusion criteria: negative (score 2) as on Frankl's behaviour rating scale
N= 30
Age range 3-10
Mean age (SD) in years, gender
Group 1 5.4 (1.81), 5 males and 10 females
Group 2 5.9 (1.67), 9 males and 6 females

Interventions
Group 1 (n=15): midazolam (0.25 mg/kg) and ketamine (3mg/kg) Oral
Group 2 (n=15): nitrous oxide-oxygen up to 50% Inhalation

Outcomes
Houpt

Bias	Authors' judgement	Support for Judgement
Random sequence generation (selection bias)	Unclear risk	Not described
Allocation concealment (selection bias)	High risk	No concealment
Blinding - Participant	High risk	No blinding
Blinding - Operator/sedationist	High risk	No blinding
Blinding - Outcome assessor	High risk	No blinding
Incomplete outcome assessment	Low risk	All randomised participants included in outcome assessment
Free of selective reporting	Low risk	All planned outcomes reported
Free of other bias	Unclear risk	Difference in gender balance at baseline (not statistically tested)

Jaikaria 2018

Parallel design
Funding: None
Location: India

Participants
Inclusion criteria: negative as on Frankl's behaviour rating scale
N= 34
Age range 3-9
Mean age (SD) in years, weight (SD) in kg
4.59 (1.20), 15.26 (2.41)

Interventions
Group 1: 0.3 mg/kg midazolam + 5 mg/kg ketamine
Group 2: 2 ug/kg dexmedetomidine + 3 ug/kg fentanyl
Group 3: 2 ug/kg dexmedetomidine + 5 mg/kg of ketamine
All Oral

Outcomes
Modified from Observer Assessment of Alertness and Sedation

Bias	Authors' judgement	Support for Judgement
Random sequence generation (selection bias)	Low risk	Envelope
Allocation concealment (selection bias)	Low risk	Described clearly
Blinding - Participant	Low risk	Blinded
Blinding - Operator/sedationist	Low risk	Dentist blinded
Blinding - Outcome assessor	Low risk	Observer blinded
Incomplete outcome assessment	Low risk	All randomised participants included in outcome assessment
Free of selective reporting	Low risk	All planned outcomes reported
Free of other bias	Low risk	No apparent differences between groups at baseline

Patel 2018

Parallel design
Funding: None
Location: India

Participants
Inclusion criteria: basic behavior modification techniques were unsuccessful
N= 44
Age range 4-9
No further information on demographics provided

Interventions
Group 1: 2 ug/kg dexmedetomidine
Group 2: 2.5 ug/kg dexmedetomidine
All intranasal

Group 3: 4 ug/kg dexmedetomidine
Group 4: 5 ug/kg dexmedetomidine
All Oral

Outcomes
Ohio State University Behavioral Rating Scale

Bias	Authors' judgement	Support for Judgement
Random sequence generation (selection bias)	High risk	No detail provided
Allocation concealment (selection bias)	High risk	No concealment
Blinding - Participant	High risk	Not blinded
Blinding - Operator/sedationist	High risk	Not blinded
Blinding - Outcome assessor	High risk	Not blinded
Incomplete outcome assessment	Low risk	All randomised participants included in outcome assessment
Free of selective reporting	Low risk	All planned outcomes reported
Free of other bias	Unclear risk	No information provided

Sado-Filho 2019

Parallel design
Funding: Conselho Nacional de Desenvolvimento Cientifico e Tecnoló gico
Location: Brazil

Participants
Inclusion criteria: uncooperative behaviour confirmed during dental exam
N= 84
Age range < 7
Mean age (range) in months, gender, weight (range) in kg
Group 1 43.5 (33 – 52.3), 14 males and 14 females, 15.7 (13.5=17.5)
Group 2 38 (33.3 - 48), 15 males and 13 females, 14.5 (13 – 17.4)
Group 3 42.5 (35 – 47.5), 14 males and 14 females, 15.4 (14.2 – 17.3)

Interventions
Group 1 (n=28): 4.0 mg/kg ketamine + 0.2 mg/kg midazolam Intranasal
Group 2 (n=28): 4.0 mg/kg ketamine + 0.5 mg/kg midazolam Oral
Group 3 (n=28): 1 mg/kg midazolam Oral

Outcomes
Ohio State University Behavioral Rating Scale

Bias	Authors' judgement	Support for Judgement
Random sequence generation (selection bias)	Low risk	Online
Allocation concealment (selection bias)	Low risk	Carried out by anaesthesiologist using envelopes
Blinding - Participant	Low risk	Blinded
Blinding - Operator/sedationist	Low risk	Blinded
Blinding - Outcome assessor	Low risk	Blinded and multiple outcome assessors used
Incomplete outcome assessment	Low risk	All randomised participants included in outcome assessment
Free of selective reporting	Low risk	All planned outcomes reported
Free of other bias	Low risk	No apparent differences between groups at baseline

Yalcin 2018

Parallel design
Funding: None
Location: Turkey

Participants
Inclusion criteria: failed to start dental treatment despite behavioral guidance techniques
N= 75
Age range 6-12
No further information on demographics provided

Interventions
Group 1 (n=25): 1 mg/kg bolus dose Ketamine followed by 50-60 µg/ kg/min continuous infusion
Group 2 (n=25): 2 mg/kg bolus dose Propofol followed by 70-90 µg/kg/min continuous infusion
Group 3 (n=25): 0.6 mg/kg bolus dose Propofol/Ketamine (1:1 mix) followed by 40-60 µg/kg/min continuous infusion
All IV

Outcomes
BIS monitoring, Observer's Assessment of Alertness/Sedation (OAAS) and Ramsay Sedation Scales (RSS) scores.

Bias	Authors' judgement	Support for Judgement
Random sequence generation (selection bias)	Low risk	Closed envelope
Allocation concealment (selection bias)	Unclear	Not described
Blinding - Participant	Low risk	Blinded
Blinding - Operator/sedationist	Low risk	Blinded
Blinding - Outcome assessor	Low risk	Blinded and multiple outcome assessors used
Incomplete outcome assessment	Low risk	All randomised participants included in outcome assessment
Free of selective reporting	Low risk	All planned outcomes reported
Free of other bias	Low risk	No differences reported between groups at baseline

Supplement 3 Frequency of studies in which drug regimens were tested

Drug regimen	Study
Dexmedetomidine	Hammadyeh 2019, Patel 2018
Dexmedetomidine and fentanyl	Jaikaria 2018
Dexmedetomidine and midazolam	Jaikaria 2018
Ketamine	Yalcin 2018
Ketamine and atropine	Hammadyeh 2019
Ketamine and propofol	Yalcin 2018
Midazolam (oral)	Sado-Filho 2019
Midazolam and ketamine (intranasal)	Sado-Filho 2019
Midazolam and ketamine (oral)	Ilasrinivasan 2018, Jaikaria 2018, Sado-Filho 2019
Nitrous oxide	Ilasrinivasan 2018
Propofol	Yalcin 2018

Supplement 4 Risk of Bias assessments of included studies from updated search

	Random sequence generation	Allocation concealment	Blinding-Participant	Blinding – operator	Blinding – outcome assessor	Incomplete outcome assessment	Free of selective reporting	Free of other bias
**Hammadyeh 2019**	+	-	-	-	?	+	+	?
**Ilasrinivasan 2018**	?	-	-	-	-	+	+	?
**Jaikaria 2018**	+	+	+	+	+	+	+	+
**Patel 2018**	-	-	-	-	-	+	+	?
**Sado-Filho 2019**	+	+	+	+	+	+	+	+
**Yalcin 2018**	+	?	+	+	+	+	+	+

Supplement 5 Behaviour and sedation rating scales from the updated search.

Rating scale	Study
Houpt	Ilasrinivasan 2018
Modified from Observer Assessment of Alertness and Sedation	Jaikaria 2018
Observer Assessment of Alertness and Sedation	Yalcin 2018
Ohio State University Behavioral Rating Scale	Hammadyeh 2019, Patel 2018, Sado-Filho 2019
Ramsay Sedation Scale	Yalcin 2018

Supplement 6 Dosage and drug comparison studies

Dosage study outcomes

Dexmedetomidine vs

Study ID	Demographics	Groups	Outcome measures	Results	Treatment completed
Patel 2018	N= 44<br>Age range 4-9<br>No further information on demographics provided	Group 1: 2 ug/kg dexmedetomidine<br>Group 2: 2.5 ug/kg dexmedetomidine<br>All intranasal<br><br>Group 3: 4 ug/kg dexmedetomidine<br>Group 4: 5 ug/kg dexmedetomidine<br>All Oral	Ohio State University Behavioural Rating Scale	Significantly better behaviour in Groups 1 and 2 compared to 3 and 4<br><br>Data reported as mean (SD)<br><br>Adverse effects: no serious side effects reported<br><br>Monitoring: cardiovascular and respiratory monitoring	Successful completion Group 1 73%, Group 2 45%, 0 in Groups 3 and 4

Comparison study outcomes

Dexmedetomidine vs

Study ID	Demographics	Groups	Outcome measures	Results	Treatment completed
Hammadyeh 2019	N= 40<br>Age range 2-6<br>Mean age (SD) in years, gender, weight (SD) in kg<br>Group 1 9.1 (0.9), 10 males and 10 females, 14.9 (9.1)<br>Group 2 8.9 (0.7), 9 males and 11 females, 15.2 (8.2)	Group 1 (n=20): dexmedetomidine loading dose of 1 µg/kg over 10 min, followed by 0.2 µg/kg as continuous infusion until desired level of sedation achieved<br>Group 2 (n=20): ketamine hydrochloride 2 mg/kg + atropine 0.01 mg/kg<br>All IV	Ohio State University Behavioral Rating Scale	Significantly better behaviour in dexmedetomidine group (p=0.03)<br><br>Data reported as mean (SD)<br><br>Adverse effects: no serious side effects reported<br><br>Monitoring: cardiovascular and respiratory monitoring	All participants completed treatment

Ketamine vs

Study ID	Demographics	Groups	Outcome measures	Results	Treatment completed
Yalcin 2018	N= 75<br>Age range 6-12	Group 1 (n=25): 1 mg/kg bolus dose Ketamine followed	BIS monitoring<br>Observer's Assessment of	Mean BIS scores at all time points	Not reported

	No further information on demographics provided	by 50-60 µg/ kg/min continuous infusion<br>Group 2 (n=25): 2 mg/kg bolus dose Propofol followed by 70-90 µg/kg/min continuous infusion<br>Group 3 (n=25): 0.6 mg/kg bolus dose Propofol/Ketamine (1:1 mix) followed by 40-60 µg/kg/min continuous infusion<br>All IV	Alertness/Sedation (OAAS)<br>Ramsay Sedation Scales (RSS) scores<br>Post-op anxiety as measured by MCDASf and CFSS DS	after drug administration in Group 2 and 2 were significantly lower than those in Group 1 ($p < 0.0001$, all time points<br><br>Mean Ramsay Sedation Scale (RSS) scores in Group 2 at all time points were significantly lower than those in Group 1 ($p < 0.0001$; $p = 0.001$; $p < 0.0001$; $p < 0.0001$) while in Group 3 mean RSS scores at 10th, 15th and 20th	

				minutes were significantly lower than those in Group 1 (p = 0.003; p = 0.002; p = 0.007).<br><br>Mean OAAS scores in Group P at all time points – except 5th minutes- and in Group KP –except 5th and 10th minutes- were significantly higher than those in Group K (p < 0.0001; p = 0.002; p < 0.0001; p < 0.0001 and p = 0.016; p < 0.0001; p < 0.0001 respectively)	

				Mean CFSS-DS and MCDASf scores after dental treatment in Group 2 and 3 significantly lower than that in Group 1 (p = 0.001, p = 0.021 and p = 0.003; p = 0.033 respectively) Medians and means presented graphically<br><br>Adverse effects: Higher in Group 1 compared to groups 2 and 3<br><br>Monitoring: blood pressure,	

				heart rate, oxygen saturation	

Midazolam and ketamine vs

Study ID	Demographics	Groups	Outcome measures	Results	Treatment completed
Ilasrinivasan 2018	N= 30<br>Age range 3-10<br>Mean age (SD) in years, gender<br>Group 1 5.4 (1.81), 5 males and 10 females<br>Group 2 5.9 (1.67), 9 males and 6 females	Group 1 (n=15): midazolam (0.25 mg/kg) and ketamine (3mg/kg) Oral<br>Group 2 (n=15): nitrous oxide-oxygen up to 50% Inhalation	Houpt	No statistical difference in MOAAS<br><br>Data reported as percentages<br><br>Adverse effects: hallucination and oversleep the following night in group 1<br><br>Monitoring: blood pressure, heart rate, oxygen saturation	Completed in 80% group 1 and 73% group 2. Not statistically significant
Jaikaria 2018	N= 34<br>Age range 3-9	Group 1: 0.3 mg/kg midazolam	Modified from Observer	No statistical difference in MOAAS	All participants

	Mean age (SD) in years, weight (SD) in kg<br>4.59 (1.20), 15.26 (2.41)	+ 5 mg/kg ketamine<br>Group 2: 2 ug/kg dexmedetomidine + 3 ug/kg fentanyl<br>Group 3: 2 ug/kg dexmedetomidine + 5 mg/kg of ketamine<br>All Oral	Assessment of Alertness and Sedation	Mean score of outcome reported<br><br>Adverse effects: Post-operative nausea and vomiting reported in all groups<br><br>Monitoring: blood pressure, heart rate, oxygen saturation	completed treatment
Sado-Filho 2019	N= 84<br>Age range < 7<br>Mean age (range) in months, gender, weight (range) in kg<br>Group 1 43.5 (33 – 52.3), 14 males and 14 females, 15.7 (13.5=17.5)	Group 1 (n=28): 4.0 mg/kg ketamine + 0.2 mg/kg midazolam Intranasal<br>Group 2 (n=28): 4.0 mg/kg ketamine + 0.5 mg/kg midazolam Oral<br>Group 3 (n=28): 1 mg/kg midazolam Oral	Ohio State University Behavioral Rating Scale	No difference in groups as assessed by dichotomous variable ‘quiet behavior for at least 60% of the session length’ was: Group 1 50.0%, Group 2 46.4%, Group 3 32.1% (P = 0.360)	Treatment completed 92.9% Group 1, 89.3% Group 2 and 85.7% Group 3

	Group 2 38 (33.3 - 48), 15 males and 13 females, 14.5 (13 – 17.4)<br>Group 3 42.5 (35 – 47.5), 14 males and 14 females, 15.4 (14.2 – 17.3)			Medians and means presented graphically<br><br>Adverse effects: Vomiting reported in all groups<br><br>Monitoring: blood pressure, heart rate, oxygen saturation	
